# Spatial mapping with Gaussian processes and nonstationary Fourier features

**DOI:** 10.1016/j.spasta.2018.02.002

**Published:** 2018-12

**Authors:** Jean-Francois Ton, Seth Flaxman, Dino Sejdinovic, Samir Bhatt

**Affiliations:** a Department of Statistics, University of Oxford, Oxford, OX1 3LB, UK; b Department of Mathematics and Data Science Institute, Imperial College London, London, SW7 2AZ, UK; c Department of Infectious Disease Epidemiology, Imperial College London, London, W2 1PG, UK

**Keywords:** Gaussian process, Nonstationary, Spatial statistics, Random Fourier features

## Abstract

The use of covariance kernels is ubiquitous in the field of spatial statistics. Kernels allow data to be mapped into high-dimensional feature spaces and can thus extend simple linear additive methods to nonlinear methods with higher order interactions. However, until recently, there has been a strong reliance on a limited class of stationary kernels such as the Matérn or squared exponential, limiting the expressiveness of these modelling approaches. Recent machine learning research has focused on spectral representations to model arbitrary stationary kernels and introduced more general representations that include classes of nonstationary kernels. In this paper, we exploit the connections between Fourier feature representations, Gaussian processes and neural networks to generalise previous approaches and develop a simple and efficient framework to learn arbitrarily complex nonstationary kernel functions directly from the data, while taking care to avoid overfitting using state-of-the-art methods from deep learning. We highlight the very broad array of kernel classes that could be created within this framework. We apply this to a time series dataset and a remote sensing problem involving land surface temperature in Eastern Africa. We show that without increasing the computational or storage complexity, nonstationary kernels can be used to improve generalisation performance and provide more interpretable results.

## 1 Introduction

The past decade has seen a tremendous and ubiquitous growth in both data collection and computational resources available for data analysis. In particular, spatiotemporal modelling has been brought to the forefront with applications in finance (Pace et al., 2000), weather forecasting (Berrocal et al., 2007), remote sensing Wan et al., 2002, Weiss et al., 2014a, Weiss et al., 2014b, Weiss et al., 2015 and demographic/disease mapping Bhatt et al., 2013, Bhatt et al., 2015, Hay et al., 2013. The methodological workhorse of mapping efforts has been Gaussian process (GP) regression Rasmussen and Williams, 2006, Diggle and Ribeiro, 2007. The reliance on GPs stems from their convenient mathematical framework which allows the modelling of distributions over nonlinear functions. GPs offer robustness to overfitting, a principled way to integrate over hyperparameters, and provide uncertainty intervals. In a GP, every point in some continuous input space is associated with a normally distributed random variable, such that every finite collection of those random variables has a multivariate normal distribution — entirely defined through a mean function μ(⋅) and a covariance kernel function k(⋅,⋅). In many settings, μ(⋅)=0 and modelling proceeds through selecting the appropriate kernel function which entirely determines the properties of the GP, and can have a significant influence on both the predictive performance and on the model interpretability Paciorek and Schervish, 2006, Genton et al., 2001. However, in practice, the kernel function is often (somewhat arbitrarily) set *a priori* to the squared exponential or Matérn class of kernels (Genton et al., 2001), justifying this choice by the fact that they model a rich class of functions (Micchelli et al., 2006).

While offering an elegant mathematical framework, performing inference with GP models is computationally demanding. Namely, evaluating the GP posterior involves a matrix inversion, which for n observations, requires $O\left(n^{3}\right)$ time and $O\left(n^{2}\right)$ storage complexity. This makes fitting full GP models prohibitive for any dataset that exceeds a few thousand observations (thereby limiting their use exactly in the regimes where a flexible nonlinear model is of interest). In response to these limitations, many scalable approximations to GP modelling have been proposed. Very broadly these can be catagorised into (a) low rank approximations, (b) sparse approximation methods and (c) spectral methods. Low rank approximations typically decompose the covariance matrix into a smaller rank m matrix to reduce the computational complexity to $O\left(nm^{2}\right)$. Popular examples include inducing points representations (Quiñonero-Candela and Rasmussen, 2005), the Nyström approximation (Rasmussen and Williams, 2006), the fully independent training conditional (FITC) model (Snelson and Ghahramani, 2012), fixed rank Kriging Cressie and Johannesson, 2008, Kang and Cressie, 2011, process convolutions (Higdon, 2002), empirical orthogonal decompositions Obled and Creutin, 1986, Wikle and Cressie, 1999 and the multi-resolution approximation (Katzfuss, 2017). Sparse approximation methods, move away from using global basis functions as in the low rank approaches and focus on compactly supported covariance representations. Popular examples include covariance tapering (Furrer et al., 2006), solutions to stochastic partial differential equations (Lindgren et al., 2011), Gauss Markov random field approximations (Rue and Tjelmeland, 2002), and nearest neighbour Gaussian processes (Datta et al., 2016). Spectral methods appeal to spectral constructions of the covariance matrix, with popular examples including spectral and multiresolution representations (Wikle et al., 2001), random Fourier features Rahimi and Recht, 2008a, Lázaro-Gredilla et al., 2010, generalised spectral kernels Samo and Roberts, 2015, Remes et al., 2017.

In this contribution, we will focus on large-scale Fourier representations of GPs. The random Fourier feature (RFF) approach RFFs implement an extremely simple, yet efficient idea: instead of relying on the implicit feature map associated with the kernel RFFs create an explicit, low-dimensional random Fourier feature map, obtained by estimating an empirical characteristic function (as opposed to common empirical orthogonal decompositions (Obled and Creutin, 1986) solving the eigen value problem) from a given spectral density Feuerverger and Mureika, 1977, Sriperumbudur and Szabo, 2015. The advantage of this explicit low-dimensional feature representation is that, unlike low rank matrix approximations, it approximates the entire kernel function not just the kernel matrix. Through numerical experiments, it has also been demonstrated that kernel algorithms constructed using the approximate kernel do not suffer from significant performance degradation Rahimi and Recht, 2008a, Rahimi and Recht, 2008b, Rahimi and Recht, 2009. To provide some intuition to the reader we perform a simple simulation experiment in Appendix. For a more thorough treatment of the theoretical properties of RFF kernels, finite-sample performance, uniform convergence bounds, and kernel approximation quality the reader is directed here Sriperumbudur and Szabo, 2015, Li and Honorio, 2017, Rahimi and Recht, 2009, Rahimi and Recht, 2008a, Rahimi and Recht, 2008b, Avron et al., 2017.

Large-scale Fourier representations of GPs traditionally rely on strong assumptions of the stationarity (or shift-invariance) of kernel functions, which is made in the vast majority of applications (and is indeed satisfied by the most often used squared exponential and Matérn kernels). Stationarity in the spatio-temporal data means that the similarity between two responses in space and time does not depend on the location and time itself, but only on the difference (or lag) between them, i.e. kernel function can be written as $k\left(x_{1},x_{2}\right)=\kappa \left(x_{1}-x_{2}\right)$ for some function κ. Several recent works Lázaro-Gredilla et al., 2010, Wilson and Adams, 2013, Yang et al., 2015 consider flexible families of kernels based on Fourier representations, thus avoiding the need to choose a specific kernel a priori and allowing the kernel to be learned from the data, but these approaches are restricted to the stationary case. In many applications, particularly when data is rich, relaxing the assumption of stationarity can greatly improve generalisation performance (Paciorek and Schervish, 2006). To address this, recent work in Samo and Roberts (2015) and Genton et al. (2001) note that a more general spectral characterisation exists that includes nonstationary kernels Yaglom, 1987, Genton et al., 2001 and uses it to construct nonstationary kernel families. In this paper, we build on the work of Samo and Roberts (2015), Lázaro-Gredilla et al. (2010), Remes et al. (2017) and develop a simple and practicable framework for learning spatiotemporal nonstationary kernel functions directly from the data by exploiting the connections between Fourier feature representations, Gaussian processes and neural networks (Rasmussen and Williams, 2006). Specifically, we directly learn frequencies in nonstationary spectral kernel representations using an appropriate neural network architecture, and adopt techniques used for deep learning regularisation (Srivastava et al., 2014) to prevent overfitting. We demonstrate the utility of the proposed method for learning nonstationary kernel functions in a time series example and in spatial mapping of land surface temperature in East Africa.

## 2 Methods and theory

### 2.1 Gaussian process regression

Gaussian process regression (GPR) takes a training dataset $D={\left\{\left(x_{i},y_{i}\right)\right\}}_{i=1}^{n}$ where $y_{i}\in R$ is real-valued response/output and $x_{i}\in R^{D}$ is a D-dimensional input vector. The response $y_{i}$ and the input $x_{i}$ are connected via the observation model

(1) $$y_{i}=f\left(x_{i}\right)+\epsilon_{i},\;\epsilon_{i}\overset{i.i.d.}{∼}N\left(0,\sigma_{n}^{2}\right),\;i=1,\ldots ,n.$$

GPR is a Bayesian non-parametric approach that imposes a prior distribution on functions f, namely a GP prior, such that any vector $f=\left[f\left(x_{1}\right),\ldots ,f\left(x_{m}\right)\right]$ of a finite number of evaluations of f follows a multivariate normal distribution $f∼N\left(0,K_{xx}\right)$, where the covariance matrix $K_{xx}$ is created as a Gram matrix based on the kernel function evaluations, ${\left[K_{xx}\right]}_{ij}=k\left(x_{i},x_{j}\right)$. Throughout this paper we will assume that the mean function of the GP prior is μ=0, however, all the approaches in this paper can be easily extended to include a mean function (Bhatt et al., 2017). In stationary settings, $k\left(x_{i},x_{j}\right)=\kappa \left(x_{i}-x_{j}\right)$ for some function κ(δ). A popular choice is the automatic relevance determination (ARD) kernel (Rasmussen and Williams, 2006), given by $\kappa \left(\delta \right)=\tau^{2}\exp\left(-\delta^{⊤}\Lambda \delta \right)$ where $\tau^{2}>0$ and $\Lambda =\text{diag}\left(\lambda_{1},\ldots ,\lambda_{D}\right)$. Kernel k will typically have hyperparameters θ (e.g. $\theta =\left[\tau ,\lambda_{1},\ldots ,\lambda_{D}\right]$ for the ARD kernel) and one can thus consider a Bayesian hierarchical model:

θ∼π(θ)

$$f|\theta ∼GP\left(0,k_{\theta }\right)$$

(2) $$y_{i}|f,x_{i},\theta ∼N\left(f\left(x_{i}\right),\sigma_{n}^{2}\right),\;i=1,\ldots ,n.$$

The posterior predictive distribution is straightforward to obtain from the conditioning properties of multivariate normal distributions. For a new input $x^{*}$, we can find the posterior predictive distribution of the associated response $y^{*}$

(3) $$p\left(y^{*}|x^{*},D,\theta \right)=N\left(y^{*};\mu_{\theta },\sigma_{\theta }^{2}\right)$$

(4) $$\mu_{\theta }=k_{x^{*}x}{\left(K_{xx}+\sigma_{n}^{2}I_{n}\right)}^{-1}y$$

(5) $$\sigma_{\theta }^{2}=\sigma_{n}^{2}+k\left(x^{*},x^{*}\right)-k_{x^{*}x}{\left(K_{xx}+\sigma_{n}^{2}I_{n}\right)}^{-1}k_{xx^{*}},$$

where $k_{xx^{*}}={\left[k\left(x_{1},x^{*}\right),\ldots ,k\left(x_{n},x^{*}\right)\right]}^{⊤}$, $k_{x^{*}x}=k_{xx^{*}}^{⊤}$ and it is understood that the dependence on θ is through the kernel $k=k_{\theta }$. The computational complexity in prediction stems from the matrix inversion ${\left(K_{xx}+\sigma_{n}^{2}I_{n}\right)}^{-1}$. The marginal likelihood (also called model evidence) of the vector of outputs $y=\left[y_{1},\ldots ,y_{n}\right]$, is given by p(y|θ)=∫p(y|f,θ)p(f|θ)df, is obtained by integrating out the GP evaluations f from the likelihood of the observation model. Maximising the marginal likelihood over hyperparameters allows for automatic regularisation and hence for selecting an appropriate model complexity. For a normal observation model in (1), the log marginal likelihood is available in closed form

(6) $$\log p\left(y|\theta \right)=-\frac{n}{2}\log\left(2\pi \right)-\frac{1}{2}\left|K_{xx}+\sigma_{n}^{2}I_{n}\right|-\frac{1}{2}y^{T}{\left(K_{xx}+\sigma_{n}^{2}I_{n}\right)}^{-1}y.$$

Computing the inverse and determinant in (6) are computationally demanding — moreover, they need to be computed for every hyperparameter value θ under consideration. To allow for computational tractability, we will use an approximation of $K_{xx}$ based on Fourier features (see Sections 2.3, 2.4).

Alternative representations can easily be used such as the primal/dual representations in a closely related frequentist method, kernel ridge regression (KRR) (Hastie et al., 2009). In contrast to KRR, optimising the marginal likelihood as above retains the same computational complexity while providing uncertainty bounds and automatic regularisation without having to tune a regularisation hyperparameter. However, the maximisation problem of (6) is non-convex thereby limiting the chance of finding a global optimum, but instead relying on reasonable local optima (Rasmussen and Williams, 2006).

### 2.2 Random Fourier feature mappings

The Wiener–Khintchine theorem states that the power spectrum and the autocorrelation function of a random process constitute a Fourier pair. Given this, random Fourier feature mappings and similar methodologies Remes et al., 2017, Yang et al., 2015, Samo and Roberts, 2015, Lázaro-Gredilla et al., 2010, Rahimi and Recht, 2008a appeal to Bochner’s theorem to reformulate the kernel function in terms of its spectral density.

Theorem 1 **Bochner’s Theorem**

*A stationary continuous kernel* $k\left(x_{i},x_{j}\right)=\kappa \left(x_{i}-x_{j}\right)$ *on* $R^{d}$ *is positive definite if and only if* κ(δ) *is the Fourier transform of a non-negative measure.*

Hence, for an appropriately scaled shift invariant complex kernel κ(δ), i.e. for κ(0)=1, Bochner’s Theorem ensures that its inverse Fourier Transform is a probability measure:

(7) $$k\left(x_{1},x_{2}\right)=\int_{R^{d}}e^{i\omega^{T}\left(x_{1}-x_{2}\right)}P\left(d\omega \right).$$

Thus, Bochner’s Theorem introduces the duality between stationary kernels and the spectral measures P(dω). Note that the scale parameter of the kernel, i.e. $\sigma_{f}^{2}=\kappa \left(0\right)$ can be trivially added back into the kernel construction by rescaling. Table 1 shows some popular kernel functions and their respective spectral densities.

By taking the real part of Eq. (7) (since we are commonly interested only in real-valued kernels in the context of GP modelling) and performing standard Monte Carlo integration, we can derive a finite-dimensional, reduced rank approximation of the kernel function

(8) $$k\left(x_{1},x_{2}\right)=\int_{R^{D}}e^{i\omega^{T}\left(x_{1}-x_{2}\right)}P\left(d\omega \right)$$

(9) $$=E_{\omega ∼P}\left[e^{i\omega^{T}\left(x_{1}-x_{2}\right)}\right],$$

(10) $$=E_{\omega ∼P}\left[\cos\left(\omega^{T}\left(x_{1}-x_{2}\right)\right)+i\sin\left(\omega^{T}\left(x_{1}-x_{2}\right)\right)\right]$$

(11) $$=E_{\omega ∼P}\left[\cos\left(\omega^{T}\left(x_{1}-x_{2}\right)\right)\right]$$

(12) $$=E_{\omega ∼P}\left[\cos\left(\omega^{T}x_{1}\right)\cos\left(\omega^{T}x_{2}\right)+\sin\left(\omega^{T}x_{1}\right)\sin\left(\omega^{T}x_{2}\right)\right]$$

(13) $$\approx \frac{1}{m}\overset{m}{\underset{k=1}{\sum }}\left(\cos\left(\omega_{k}^{T}x_{1}\right)\cos\left(\omega_{k}^{T}x_{2}\right)+\sin\left(\omega_{k}^{T}x_{1}\right)\sin\left(\omega_{k}^{T}x_{2}\right)\right)$$

(14) $$=\frac{1}{m}\overset{m}{\underset{k=1}{\sum }}\Phi_{k}{\left(x_{1}\right)}^{T}\Phi_{k}\left(x_{2}\right)$$

where ${\left\{\omega_{k}\right\}}_{k=1}^{m}\overset{i.i.d.}{∼}P$ and we denoted

$$\Phi_{k}\left(x_{l}\right)=\left(\begin{array}{c} \cos\left(\omega_{k}^{T}x_{l}\right)\\ \sin\left(\omega_{k}^{T}x_{l}\right) \end{array}\right).$$

**Table 1:** Summary table of kernels and their spectral densities.

Kernel name	k(δ)	p(ω)
Squared exponential	$e^{-\frac{\left({\| \delta \|}_{2}^{2}\right)}{2\sigma }}$, σ>0	${\left(2\pi \right)}^{-\frac{D}{2}}\sigma^{D}\exp\left(-\frac{\sigma^{2}{\| \omega \|}_{2}^{2}}{2}\right)$
Laplacian	$\exp\left(-\sigma \|\delta {\|}_{1}\right)$, σ>0	${\left(\frac{2}{\pi }\right)}^{\frac{2}{D}}\prod_{i=1}^{D}\frac{\sigma }{\sigma^{2}+\omega_{i}^{2}}$
Matérn	$\frac{2^{1-\lambda }}{\Gamma \left(\lambda \right)}{\left(\frac{\sqrt{\left(2\lambda \right)\|\delta {\|}_{2}}}{\sigma }\right)}^{\lambda }K_{\lambda }\left(\frac{\sqrt{\left(2\lambda \right)\|\delta {\|}_{2}}}{\sigma }\right)$	$\frac{2^{D+\lambda }\pi^{\frac{D}{2}}\Gamma \left(\lambda +D∕2\right)\lambda^{\lambda }}{\Gamma \left(\lambda \right)\sigma^{2\lambda }}{\left(\frac{2\lambda }{\sigma^{2}}+4\pi^{2}{\| \omega \|}_{2}^{2}\right)}^{-\left(\lambda +D∕2\right)}$
	λ>0,σ>0	

For a covariate design matrix $X\in R^{n\times D}$ (with rows corresponding to data vectors $x_{1},\ldots ,x_{n}$), and frequency matrix $\Omega \in R^{m\times D}$ (with rows corresponding to frequencies $\omega_{1},\ldots ,\omega_{m}$), we let $\Phi_{x}=\left[\cos\left(X\Omega^{⊤}\right)\;\sin\left(X\Omega^{⊤}\right)\right]$ be a n×2m matrix referred to as the feature map of the dataset. The estimated covariance matrix can be computed as Kxx^=1mΦxΦxT which has rank at most 2m. Substituting Kxx^ into (6) now allows rewriting the determinant and the inverse in terms of the 2m×2m matrix ${\Phi_{x}}^{T}\Phi_{x}$, thereby reducing the computational cost of inference from $O\left(n^{3}\right)$ to $O\left(nm^{2}\right)$, where m is the number of Monte Carlo samples/frequencies. Typically, m≪n.

In particular, by defining $A={\Phi_{x}}^{T}\Phi_{x}+m\frac{\sigma_{n}^{2}}{\sigma_{f}^{2}}I_{2m}$ where $\sigma_{n}^{2}$ is the observation noise variance and $\sigma_{f}^{2}=\kappa \left(0\right)$ is the kernel scale parameter, and taking R=chol(A) to be the Cholesky factor of A, we first calculate vectors $\alpha_{1},\alpha_{2}$ solving the linear systems of equations $R\alpha_{1}={\Phi_{x}}^{T}y$, $R^{T}\alpha_{2}=\alpha_{1}$ and $R^{T}\alpha_{3}=\phi_{x^{*}}$. The log marginal likelihood can then be computed efficiently as:

(15) $$\log p\left(y|\theta \right)=-\frac{1}{2\sigma_{n}^{2}}\left(\|y{\|}^{2}-\|\alpha_{1}{\|}^{2}\right)-\frac{1}{2}\underset{i}{\sum }\log\left(R_{ii}^{2}\right)+m\log\left(m\frac{\sigma_{n}^{2}}{\sigma_{f}^{2}}\right)-\frac{n}{2}\log\left(2\pi \sigma_{n}^{2}\right).$$

Additionally, the posterior predictive mean and variance can be estimated as

(16) μθ^=σf2mΦx∗Tα2

(17) σθ2^=σn21+σf2m‖α3‖2.

There are two important disadvantages of standard random Fourier features as proposed by Rahimi and Recht (2008a): firstly, only stationary (shift invariant) kernels can be approximated, and secondly we have to select a priori a specific class of kernels and their corresponding spectral distributions (e.g. Table 1). In this paper, we address both of these limitations, with a goal to construct methods to learn a nonstationary kernel from the data, while preserving the computational efficiency of random Fourier features.

While we can think about the quantities in (16) as giving approximations to the full GP inference with a given kernel k, they are in fact performing exact GP calculations for another kernel $\overset{ˆ}{k}$ defined using the explicit feature map $\Phi_{x}$ defined through frequencies sampled from the spectral measure of k. We can thus think about these feature maps as parametrising a family of kernels in their own right and treat frequencies $\omega_{1},\ldots ,\omega_{m}$ as kernel parameters to be optimised, i.e. learned from the data by maximising the log marginal likelihood. It should be noted that dropping the imaginary part of our kernel symmetrises the spectral measure allowing us to use any P(dω) — regardless of its symmetry properties, we will still have a real-valued kernel. In particular, one can use an empirical spectral measure defined by any finite set of frequencies.

### 2.3 Nonstationary random Fourier features

Contrary to stationary kernels, which only depend on the lag vector i.e. $\delta =x_{i}-x_{j}$, nonstationary kernels depend on the inputs themselves. A simple example of a nonstationary kernel would be the polynomial kernel defined as:

(18) $$k\left(x_{1},x_{2}\right)={\left(x_{1}^{⊤}x_{2}+1\right)}^{r}.$$

To extend the stationary random feature mapping to nonstationary kernels, following Samo and Roberts (2015) Genton et al. (2001) and Yaglom (1987), we will need to use a more general spectral characterisation of positive definite functions which encompasses stationary *and* nonstationary kernels.

Theorem 2 **Yaglom, 1987; Genton et al., 2001**

*A nonstationary kernel* $k\left(x_{1},x_{2}\right)$ *is positive definite in* $R^{d}$ *if and only if it has the form:*

(19) $$k\left(x_{1},x_{2}\right)=\int_{R^{D}\times R^{D}}e^{i\left(w_{1}^{T}x_{1}-w_{2}^{T}x_{2}\right)}\mu \left(dw_{1},dw_{2}\right)$$

*where* $\mu \left(dw_{1},dw_{2}\right)$ *is the Lebesgue–Stieltjes measure associated to some positive semi-definite function* $f\left(w_{1},w_{2}\right)$ *with bounded variation.*

From the above theorem, a nonstationary kernel can be characterised by a spectral measure $\mu \left(d\omega_{1},d\omega_{2}\right)$ on the product space $R^{D}\times R^{D}$. Again, without loss of generality we can assume that μ is a probability measure. If μ is concentrated along the diagonal, $\omega_{1}=\omega_{2}$, we recover the spectral representation of stationary kernels in the previous section. However, exploiting this more general characterisation, we can construct feature mappings for nonstationary kernels.

Just like in the stationary case, we can approximate (19) using Monte Carlo integration. In order to ensure a valid positive semi-definite spectral density we first have to symmetrise $f\left(\omega_{1},\omega_{2}\right)$ by ensuring $f\left(\omega_{1},\omega_{2}\right)=f\left(\omega_{2},\omega_{1}\right)$ and including the diagonal components $f\left(\omega_{1},\omega_{1}\right)$ and $f\left(\omega_{2},\omega_{2}\right)$ (Remes et al., 2017). We can take a general form of density g on the product space and “symmetrise”:

$$f\left(\omega_{1},\omega_{2}\right)=\frac{1}{4}\left(g\left(\omega_{1},\omega_{2}\right)+g\left(\omega_{2},\omega_{1}\right)+g\left(\omega_{1},\omega_{1}\right)+g\left(\omega_{2},\omega_{2}\right)\right).$$

Once again using Monte Carlo integration we get:

$$k\left(x_{1},x_{2}\right)=\frac{1}{4}\int_{R^{D}\times R^{D}}\left(e^{i\left(\omega_{1}^{T}x_{1}-\omega_{2}^{T}x_{2}\right)}+e^{i\left(\omega_{2}^{T}x_{1}-\omega_{1}^{T}x_{2}\right)}+e^{i\left(\omega_{1}^{T}x_{1}-\omega_{1}^{T}x_{2}\right)}+e^{i\left(\omega_{2}^{T}x_{1}-\omega_{2}^{T}x_{2}\right)}\right)\mu \left(d\omega_{1}d\omega_{2}\right)=\frac{1}{4}E_{\mu }\left(e^{i\left(\omega_{1}^{T}x_{1}-\omega_{2}^{T}x_{2}\right)}+e^{i\left(\omega_{2}^{T}x_{1}-\omega_{1}^{T}x_{2}\right)}+e^{i\left(\omega_{1}^{T}x_{1}-\omega_{1}^{T}x_{2}\right)}+e^{i\left(\omega_{2}^{T}x_{1}-\omega_{2}^{T}x_{2}\right)}\right)\approx \frac{1}{4m}\overset{m}{\underset{k=1}{\sum }}\left(e^{i\left(x_{1}^{T}\omega_{k}^{1}-x_{2}^{T}\omega_{k}^{2}\right)}+e^{i\left(x_{1}^{T}\omega_{k}^{2}-x_{2}^{T}\omega_{k}^{1}\right)}+e^{i\left(x_{1}^{T}\omega_{k}^{1}-x_{2}^{T}\omega_{k}^{1}\right)}+e^{i\left(x_{1}^{T}\omega_{k}^{2}-x_{2}^{T}\omega_{k}^{2}\right)}\right)=\frac{1}{4m}\overset{m}{\underset{k=1}{\sum }}\left\{\cos\left(x_{1}^{T}\omega_{k}^{1}\right)\cos\left(x_{2}^{T}\omega_{k}^{1}\right)+\cos\left(x_{1}^{T}\omega_{k}^{1}\right)\cos\left(x_{2}^{T}\omega_{k}^{2}\right)\;\;+\cos\left(x_{1}^{T}\omega_{k}^{2}\right)\cos\left(x_{2}^{T}\omega_{k}^{1}\right)+\cos\left(x_{1}^{T}\omega_{k}^{2}\right)\cos\left(x_{2}^{T}\omega_{k}^{2}\right)\;\;+\sin\left(x_{1}^{T}\omega_{k}^{1}\right)\sin\left(x_{2}^{T}\omega_{k}^{1}\right)+\sin\left(x_{1}^{T}\omega_{k}^{1}\right)\sin\left(x_{2}^{T}\omega_{k}^{2}\right)\;\;+\sin\left(x_{1}^{T}\omega_{k}^{2}\right)\sin\left(x_{2}^{T}\omega_{k}^{1}\right)+\sin\left(x_{1}^{T}\omega_{k}^{2}\right)\sin\left(x_{2}^{T}\omega_{k}^{2}\right)\right\}\;\mathrm{(taking the real part)}=\frac{1}{4m}\overset{m}{\underset{k=1}{\sum }}\Phi_{k}{\left(x_{1}\right)}^{T}\Phi_{k}\left(x_{2}\right)$$

where ${\left\{\left(\omega_{k}^{1},\omega_{k}^{2}\right)\right\}}_{k=1}^{m}\overset{i.i.d.}{∼}\mu$ and

$$\Phi_{k}\left(x_{l}\right)=\left(\begin{array}{c} \cos\left(x_{l}^{T}\omega_{k}^{1}\right)+\cos\left(x_{l}^{T}\omega_{k}^{2}\right)\\ \sin\left(x_{l}^{T}\omega_{k}^{1}\right)+\sin\left(x_{l}^{T}\omega_{k}^{2}\right) \end{array}\right).$$

Hence, by denoting $\Omega^{l}\in R^{m\times D}$ (with rows corresponding to frequencies $\omega_{1}^{l},\ldots ,\omega_{m}^{l}$) for l=1,2 as before, we obtain the corresponding feature map for the approximated kernel as an n×2m matrix

(20) $$x\to \Phi_{x}=\left[\cos\left(X{\left(\Omega^{1}\right)}^{T}\right)+\cos\left(X{\left(\Omega^{2}\right)}^{T}\right)|\sin\left(X{\left(\Omega^{1}\right)}^{T}\right)+\sin\left(X{\left(\Omega^{2}\right)}^{T}\right)\right]$$

and can be condensed to an identical form as in the stationary case.

(21) Kxx^=14mΦxΦxT.

The non-stationarity in Eq. (21) arises from the product of differing locations $x_{1}$ and $x_{2}$ by different frequencies $\omega_{k}^{1},\omega_{k}^{2}$, hence making the kernel dependent on the values of $x_{1}$ and $x_{2}$ and not only the lag vector. If the frequencies were exactly the same we just revert to the stationary case. The complete construction of random Fourier feature approximation is summarised in the algorithm below.

**Figure:**
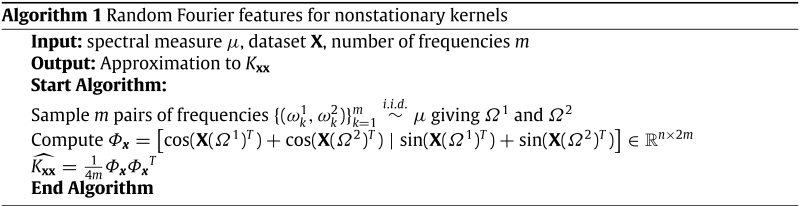

However, just like in the stationary case, we can think about nonstationary Fourier feature maps as parametrising a family of kernels and treat frequencies ${\left\{\left(\omega_{k}^{1},\omega_{k}^{2}\right)\right\}}_{k=1}^{m}$ as kernel parameters to be learned by maximising the log marginal likelihood, which is an approach we pursue in this work. Again, symmetrisation due to dropping imaginary parts implies that any empirical spectral measure is valid (there are no constraints on the frequencies).

### 2.4 On the choice of spectral measure in non-stationary case

Using the characterisation in Eq. (21) one only requires the specification of the (Lebesgue–Stieltjes measurable) distribution $f\left(\omega_{1},\omega_{2}\right)$ in order to construct a nonstationary kernel. This very general formulation allows us to create the full spectrum encompassing both simple and highly complex kernels.

In the simplest case, $f\left(\omega_{1},\omega_{2}\right)=f_{1}\left(\omega_{1}\right)f_{2}\left(\omega_{2}\right)$, i.e. it can be a product of popular spectral densities listed in Table 1. Furthermore, one could consider cases where these individual spectral densities factorise further across dimensions. This corresponds to a notion of *separability*. In spatio-temporal data, separability can be very useful as it enables interpretation of the relationship between the covariates as well as computationally efficient estimations and inferences (Finkenstadt et al., 2007). Practical implementation is straightforward; consider the classic spatio-temporal setting with 3 covariates — longitude, latitude and time. When drawing random samples of $\omega_{l}=\left(\omega_{l}^{1},\omega_{l}^{2},\omega_{l}^{3}\right)$ where l∈{1,2}, we could define the $\omega_{l}^{i}$ to come from different distributions, allowing us to individually model each input dimension. If the distribution on frequencies is independent across dimensions then we see that if $\omega_{1}=\left(\omega_{1}^{1},\omega_{1}^{2},\omega_{1}^{3}\right)$ and $\omega_{2}=\left(\omega_{2}^{1},\omega_{2}^{2},\omega_{2}^{3}\right)$:

(22) $$k\left(x_{1},x_{2}\right)=\int e^{i\omega_{1}^{T}x_{1}-i\omega_{2}^{T}x_{2}}f\left(\omega_{1},\omega_{2}\right)d\omega_{1}d\omega_{2}$$

(23) $$=\int e^{i\left(\omega_{1}^{1}x_{1}^{1}+\omega_{1}^{2}x_{1}^{2}+\omega_{1}^{3}x_{1}^{3}-\omega_{2}^{1}x_{2}^{1}-\omega_{2}^{2}x_{2}^{2}-\omega_{2}^{3}x_{2}^{3}\right)}\overset{3}{\underset{p=1}{\prod }}f\left(\omega_{1}^{p},\omega_{2}^{p}\right)d\omega_{1}^{p}d\omega_{2}^{p}$$

(24) $$=k_{1}\left(x_{1}^{1},x_{2}^{1}\right)k_{2}\left(x_{1}^{2},x_{2}^{2}\right)k_{3}\left(x_{1}^{3},x_{2}^{3}\right).$$

A practical example for spatio-temporal modelling of a nonstationary separable kernel would be generating a four dimensional ($\omega_{1}^{1},\omega_{2}^{1},\omega_{1}^{2},\omega_{2}^{2}$), sample from independent Gaussian distributions (whose spectral density corresponds to a squared exponential kernel) representing nonstationary spatial coordinates, and a two dimensional ($\omega_{1}^{3},\omega_{2}^{3}$) from a Student-t distribution with 0.5 degrees of freedom (whose spectral density corresponds to a Matérn 1∕2 kernel or exponential kernel) representing temporal coordinates.

To move from separable to non-separable, nonstationary kernels one only needs to introduce some dependence structure within $\omega^{1}$ or $\omega^{2}$ i.e. across feature dimensions, such as for example using the multivariate normal distribution in $R^{D}$, in order to prevent the factorisation in Eq. (22). The correlation structure in these multivariate distributions is what creates the non-separability.

To create non-separable kernels with different spectral densities along each feature dimension copulas can be used. An example in a spatial (latitude, longitude feature dimensions) setting using the Gaussian copula, would involve generating samples for $\omega^{1}$ or $\omega^{2}\in R^{2}$ (or both) from a multivariate normal distribution ${\left\{\omega_{k}^{1}\right\}}_{k=1}^{m}\overset{i.i.d.}{∼}N\left(0,\Sigma \right)$, pass these through the Gaussian cumulative distribution function, and then passed through the quantile function of another distribution (Λ) i.e. $C_{\Lambda }\left(\omega^{1}\right)=CDF_{\Lambda }\left(CDF_{N}^{-1}\left(\omega^{1}\right)\right)$. This transformation can also be done using different Λs along different feature dimensions. Alternative copulas can be readily used, including the popular Archimedian Copulas: Clayton, Frank and Gumbel (Genest and MacKay, 1986). Additionally, mixtures of multivariate normals can be used Remes et al., 2017, Yang et al., 2015 to create arbitrarily complex non-separable and nonstationary kernels. Given sufficient components any probability density function can be approximated to the desired accuracy.

In this paper, we focus on the most general case where the frequencies ${\left\{\left(\omega_{k}^{1},\omega_{k}^{2}\right)\right\}}_{k=1}^{m}$ are treated as kernel parameters and are learnt directly from the data by optimising the marginal likelihood, i.e. they are not associated to any specific family of joint distributions. This approach allows us to directly learn nonstationary kernels of arbitrary complexity as m increases. However, a major problem with such a heavily overparametrized kernel is the possibility of overfitting. Stationary examples of learning frequencies directly from the data Lázaro-Gredilla et al., 2010, Gal and Turner, 2015, Tan et al., 2013 have been known to overfit despite the regularisation due to working with marginal likelihood. This problem is further exacerbated in high-dimensional settings, such as those in spatio-temporal mapping with covariates. In this paper, we include an additional regularisation inspired by dropout (Srivastava et al., 2014) which prevents the co-adaptation of the learnt frequencies $\omega_{1},\omega_{2}$.

### 2.5 Gaussian dropout regularisation

Dropout (Srivastava et al., 2014) is a regularisation technique introduced to mitigate overfitting in deep neural networks. In its simplest form, dropout involves setting features/matrix entries to zero with probability q=1−p, i.e. according to a Bernoulli(p) for each feature. The main motivation behind the algorithm is to prevent co-adaptation by forcing features to be robust and rely on population behaviour. This prevents individual features from overfitting to idiosyncrasies of the data.

**Fig. 1:**
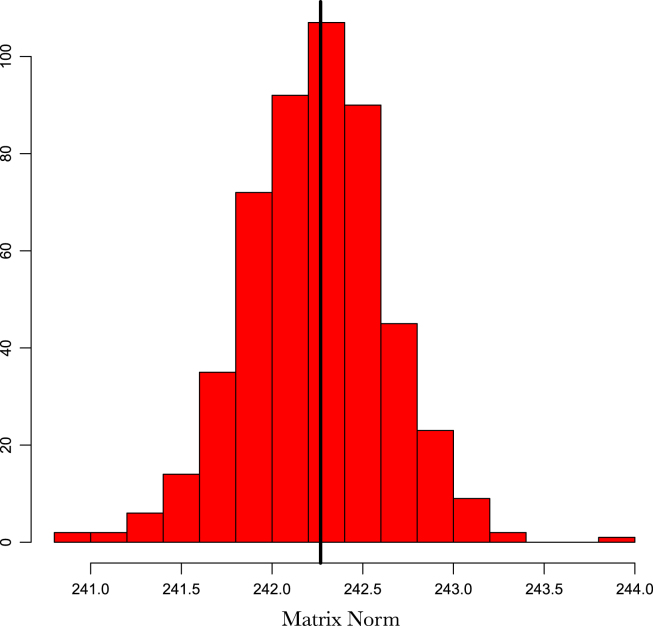
Histogram of the Euclidean norm of a covariance matrix $\Phi \Phi^{T}$ with Gaussian dropout of $\sigma_{p}=0.05$. The black line is the norm of $\Phi \Phi^{T}$*without* noise.

Using standard dropout, where zeros are introduced into the frequencies ${\left\{\left(\omega_{k}^{1},\omega_{k}^{2}\right)\right\}}_{k=1}^{m}$ can be problematic due to the trigonometric transformations in the projected features. An alternative to dropout that has been shown to be just as effective if not better is Gaussian dropout Srivastava et al., 2014, Baldi and Sadowski, 2013. Regularisation via Gaussian dropout involves augmenting our sample distribution as ${\left\{{\left(\omega_{k}^{1},\omega_{k}^{2}\right)}_{\eta }\right\}}_{k=1}^{m}=N\left(1,\sigma_{p}^{2}\right)⊙{\left\{\left(\omega_{k}^{1},\omega_{k}^{2}\right)\right\}}_{k=1}^{m}$. The addition of noise through $N\left(1,\sigma_{p}^{2}\right)$ ensures unbiased estimates of the covariance matrix i.e. $E\left[{\left\{{\left(\omega_{k}^{1},\omega_{k}^{2}\right)}_{\eta }\right\}}_{k=1}^{m}\right]=E\left[{\left\{\left(\omega_{k}^{1},\omega_{k}^{2}\right)\right\}}_{k=1}^{m}\right]$ (see Fig. 1). As with dropout, this approach prevented our population Monte Carlo sample from co-adapting, and ensured that the learnt frequencies are robust and not overfitting noise in the data. An additional benefit of this procedure over improving generalisation error and preventing overfitting was to speed up the convergence of gradient descent optimisers through escaping saddle points more effectively (Lee et al., 2017). The noise parameter $\sigma_{p}$ defines the degree of regularisation and is a hyperparameter that needs to be tuned. However, we found in practice when coupled with an early stopping procedure, learning the frequencies is robust to sensible choices of $\sigma_{p}$.

## 3 Implementation details

All of the modelling was performed within the TensorFlow framework (Abadi et al., 2016). Optimisation was performed using ADAM (Kingma and Ba, 2014) gradient descent. In addition to Gaussian dropout, additional regularisation was introduced by using early stopping (Prechelt, 1998). The early stopping criteria used followed the recommendations in Prechelt (1998), where the relative cross validation test accuracy was monitored and early stopping of the optimisation process was performed if the testing accuracy was not improved after a certain number of gradient decent steps (termed the patience parameter Prechelt, 1998). Cross-validation was performed using a 70–30 split averaged over 20 independent runs and testing performance evaluated via the mean squared error and correlation. The testing performance of the posterior predictive distribution and uncertainty was performed using the continuous ranked probability score (CRPS) (Matheson and Winkler, 1976) and the probability integral transform (PIT) (Held et al., 2010). A well calibrated model should have PIT scores distributed approximately ∼Uniform(0,1), and CRPS scores clustered towards zero.

The two hyperparameters not integrated out in the marginal likelihood: the dropout noise $\sigma_{p}$ and the gradient descent learning rate were chosen via a random search (Bergstra and Bengio, 2012) over 500 samples, and parameters selected as those returning the lowest average mean squared cross validation testing error.

To choose the number of features we follow the theoretical recommendations of Rudi and Rosasco (2016) who show that $O\left(1∕\sqrt{(}n)\right)$ generalisation learning bounds can be achieved under certain conditions with $O\left(log\left(n\right)\sqrt{(}n)\right)$ features (Rudi and Rosasco, 2016). These bounds result in the same prediction accuracy of the exact kernel ridge regression estimator. Starting from $m=log\left(n\right)\sqrt{(}n)$ features the final number of Fourier features was estimated by successively increasing m and monitoring the cross validation test accuracy. The optimal number of features was selected as those which asymptote the mean squared cross validation testing error. Code from this paper can be found at ($github.com∕bhattsamir∕Non\text{\_}stationary_{f}eatures$).

## 4 Results

### 4.1 Google daily high stock price

To demonstrate the use of the developed method and the utility of nonstationary modelling, we consider time series data of the daily high stock price of Google spanning 3295 days from 19th August 2004 to 20th September 2017. We set x∈{1,…,3295} and $y=\log\left(Stock_{high}\right)$. For the stationary case we use vanilla random Fourier features Rahimi and Recht, 2008a, Lázaro-Gredilla et al., 2010 with the squared exponential kernel (Gaussian spectral density) and m=600 *fixed* frequencies. For the nonstationary case we use m=300 frequencies for each $\omega_{1}$ and for $\omega_{2}$. We performed a sensitivity analysis to check that no improvements in either the log marginal likelihood or testing error resulted from using more features.

Fig. 2 (top left) shows the comparison in the optimisation paths of the negative log marginal likelihood between the two methods. It is clear that the nonstationary approach reaches a lower minima than the vanilla random Fourier features approach. This is also mirrored in the testing accuracy over the 20 independent runs where our approach achieves a mean squared error and correlation of $3.29\times 10^{-5}$ and 0.999, while the vanilla Fourier features approach achieves a mean squared error and correlation of $5.69\times 10^{-5}$ and 0.987. Of note is the impact of our Gaussian dropout regularisation, which, through the injection of noise, appears to converge faster and avoid plateaus. This is entirely in keeping with previous experiences using dropout variants (Baldi and Sadowski, 2013) and highlights an added benefit over using only ridge (weight decay) regularisation. Posterior predictive checks using the PIT and CRPS indicate good fits (see Fig. 4).

Fig. 3 (top) shows the overall fits compared to the raw data. Both methods appear to fit the data very well, as reflected in the testing statistics, but when examining a zoomed-in transect it is clear that the learnt nonstationary features fit the data better than the vanilla random features by allowing variable degree of smoothness in time. The combination of nonstationarity and kernel flexibility allowed us to learn a much better characterisation of the data patterns without overfitting. The covariance matrix comparisons (Fig. 3 bottom) further highlight this point where the learnt nonstationary covariance matrix shares some similarities with the vanilla random features covariance matrix, such as the concentration on the diagonal, but exhibits a much greater degree of texture. The histograms in Fig. 3 provide another perspective on the covariance structure, where the vanilla features are by design Gaussian distributed, but learnt nonstationary frequencies are far from Gaussian (Kolmogorov–Smirnov test p-value$<10^{-16}$) exhibiting skewness and heavy tails. Additionally, the differences between the learnt frequencies $\omega_{1}$ and $\omega_{2}$ show that, not only is the learnt kernel far from Gaussian, but that it is indeed also nonstationary. This simple example also highlights the potential deficiencies of choosing kernels/frequencies apriori.Fig. 2(top left) Log marginal likelihood (Y-axis), optimisation gradient update count (X-axis), vanilla random features (blue), proposed approach (red); (top right) Histogram of learnt $\omega_{1}$ for our nonstationary approach; (bottom left) Histogram of learnt $\omega_{2}$ for our nonstationary approach; (bottom right) Histogram of $\omega_{2}$ for vanilla random Fourier features. (For interpretation of the references to colour in this figure legend, the reader is referred to the web version of this article.)

**Fig. 2(a):**
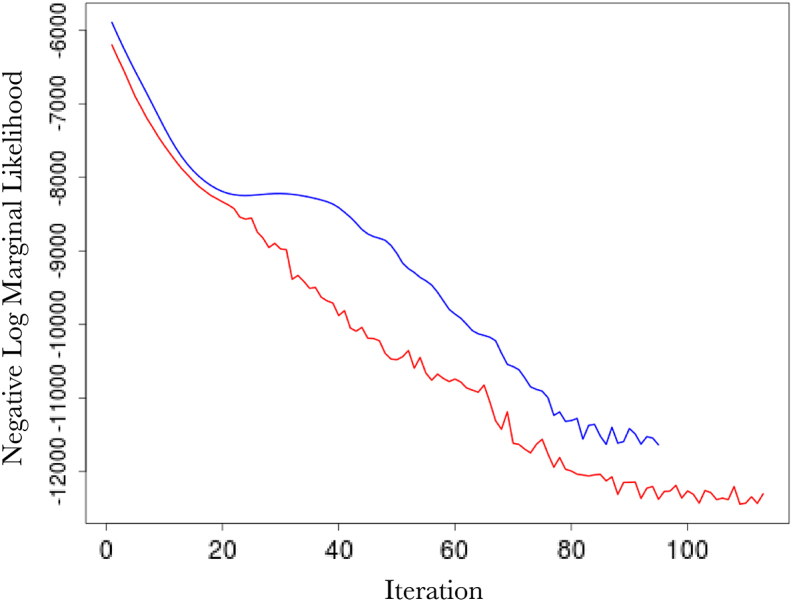
(a)

**Fig. 2(b):**
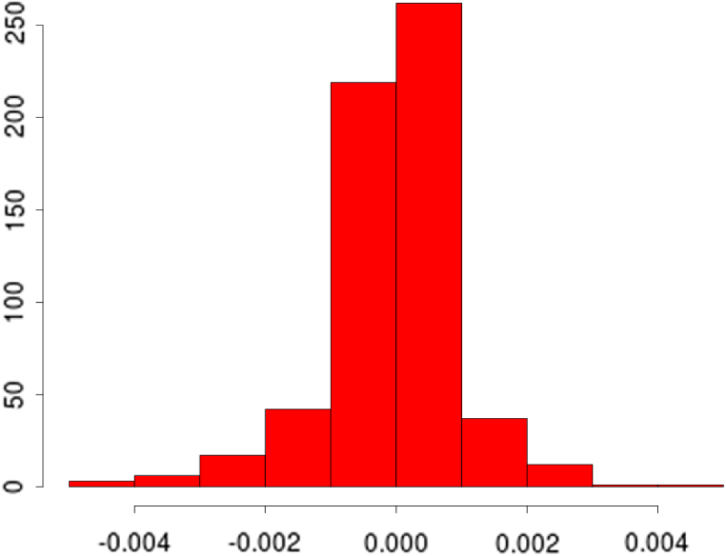
(b)

**Fig. 2(c):**
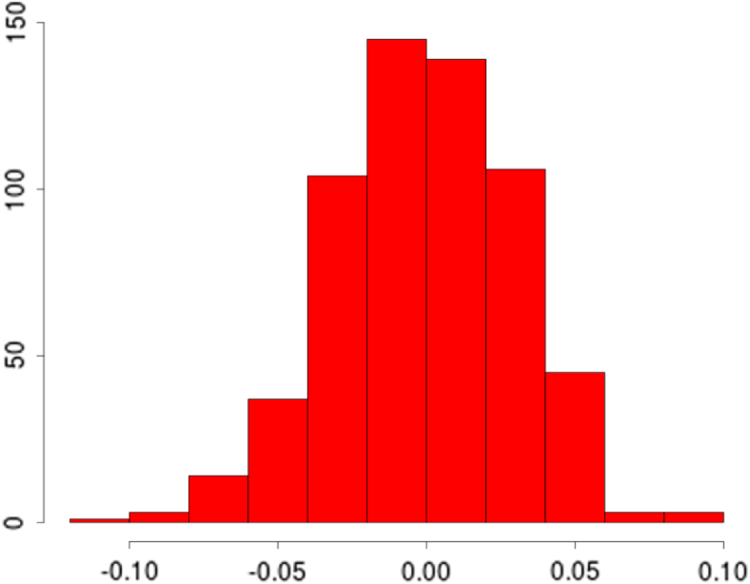
(c)

**Fig. 2(d):**
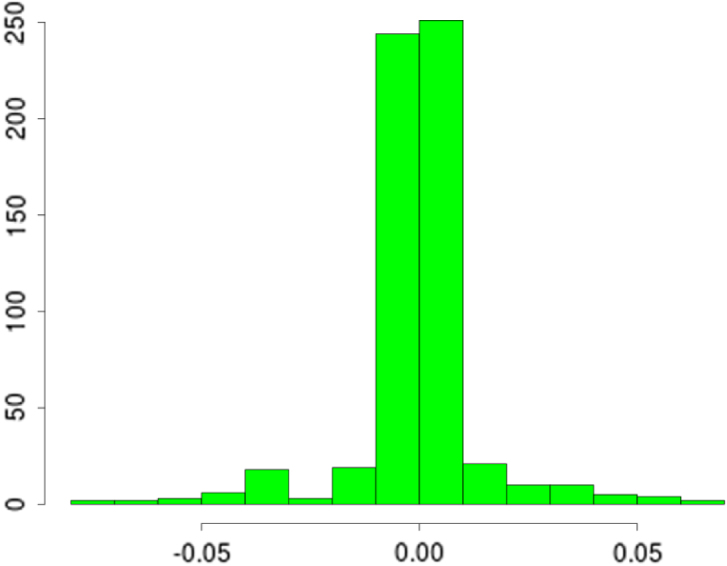
(d)

Fig. 3(top) Log daily-high Google stock price (Y-axis), days since 19th August 2004 (X-axis). Vanilla random features (blue) our proposed approach (red), actual data (black), with a zoomed in transect (purple box); (bottom left) Image of covariance matrix for our nonstationary method; (bottom right) Image of covariance matrix for vanilla random Fourier features. (For interpretation of the references to colour in this figure legend, the reader is referred to the web version of this article.)

**Fig. 3(a):**
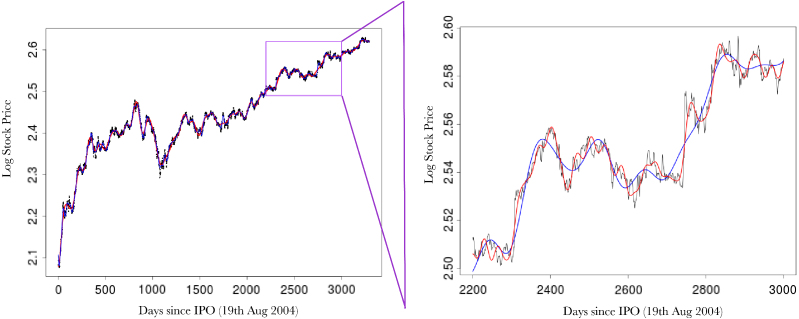
(a)

**Fig. 3(b):**
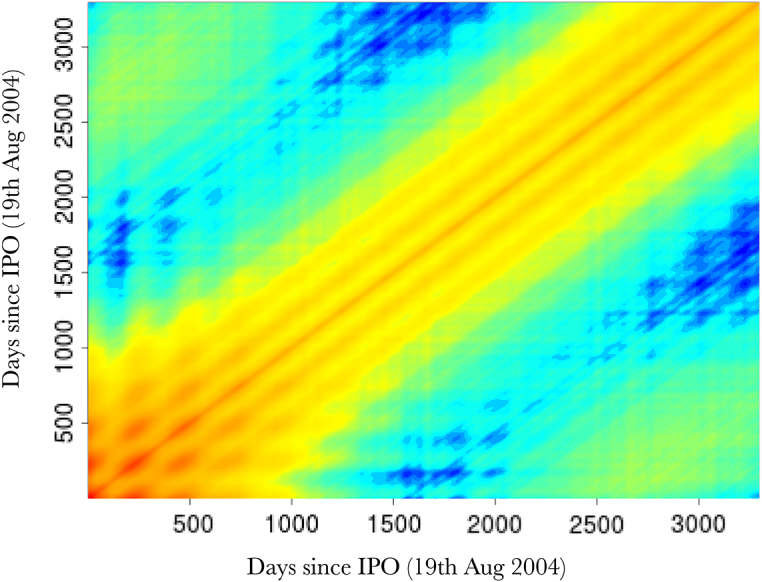
(b)

**Fig. 3(c):**
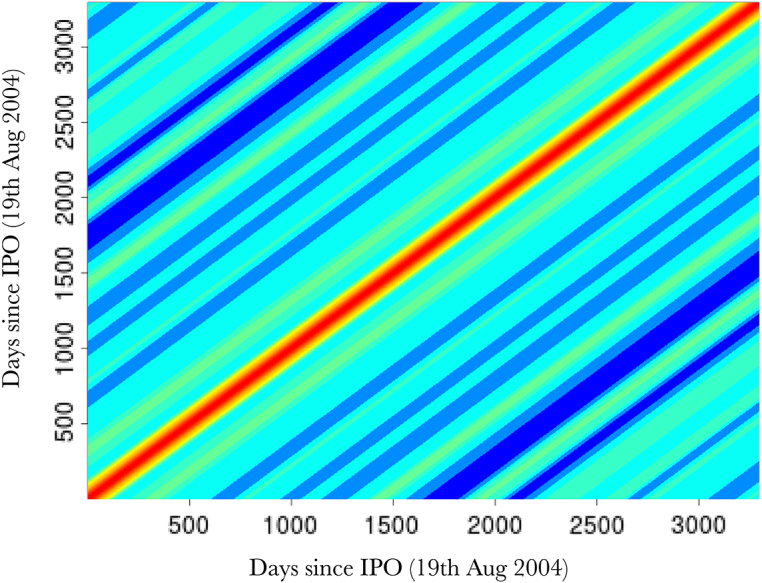
(c)

**Fig. 4:**
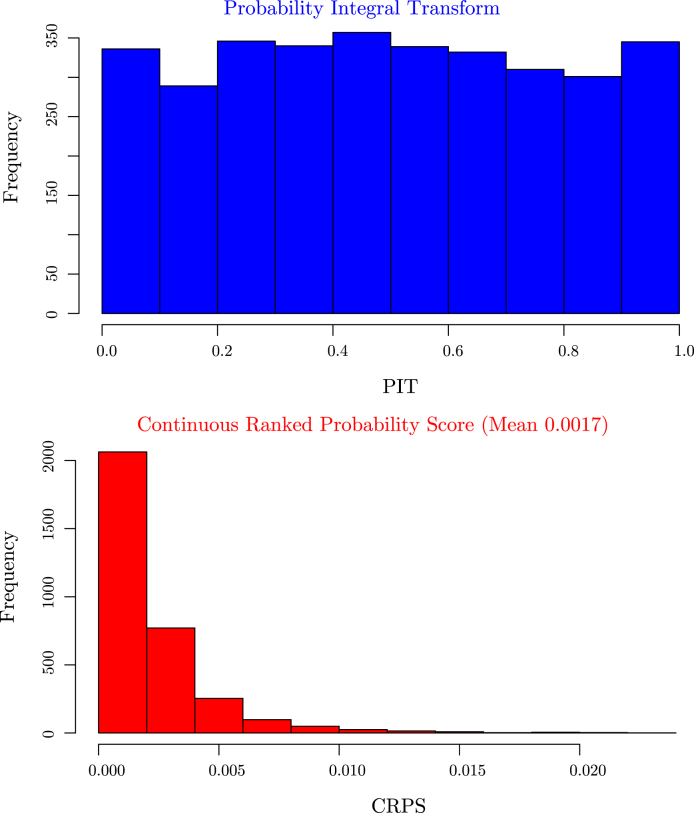
Validation summary statistics for the google time series analysis with the probability integral transform (top) and the continuous ranked probability score (bottom). Both score statistics indicate a good fit for the posterior predictive distribution.

### 4.2 Spatial temperature anomaly for East Africa in 2016

We next consider MOD11A2 Land Surface Temperature (LST) 8-day composite data (Wan et al., 2002), gap filled for cloud cover images (Weiss et al., 2014a) and averaged to a synoptic yearly mean for 2016. To replicate common situations for spatial mapping, such as interpolation from sparse remote sensed sites or cluster house hold survey locations (Bhatt et al., 2017) we randomly sample 6000 LST locations (only ∼5% of the total) from the East Africa region (see Fig. 6, Fig. 7). We set $x\in R^{2}=\left\{Latitude,Longitude\right\}$ i.e. using only the spatial coordinates as covariates, and use the LST temperature anomaly as the response. We apply our nonstationary approach, learning a total of m=1500 frequencies (750 sine and 750 cos). Cross validation was evaluated over all pixels excluding the training set (∼83,000) and averaged over 20 independent runs with testing performance evaluated via the mean squared error, correlation, CRPS and PIT. We also compare our fit to 3 of the best performing spatial statistics methods (Heaton et al., 2017) using the exact same data and using approximately the same number of basis functions. The three comparison methods were the SPDE approach (Lindgren et al., 2011), LatticeKrig (Nychka et al., 2015) and the multi-resolution approximation (MRA) (Katzfuss, 2017). Following Heaton et al. (2017) all three models were fit with a Matérn covariance function with ν=1. For the SPDE model 1539 mesh basis functions were chosen, for LatticeKrig 1534 basis functions were used (5 levels with weight 4.4 and NC = 2.15) and for MRA 1550 basis functions were used.

**Fig. 5:**
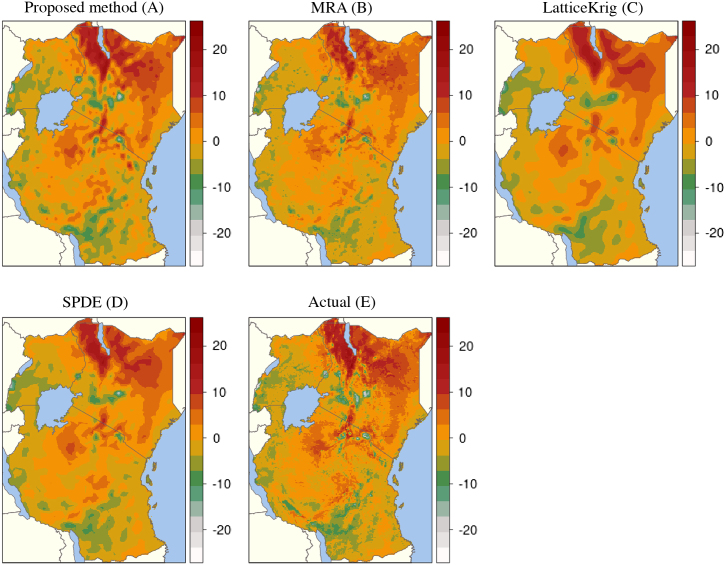
Predicted surfaces of our approach and the current state-of-the-art: The SPDE, MRA and LatticeKrig.

**Fig. 6:**
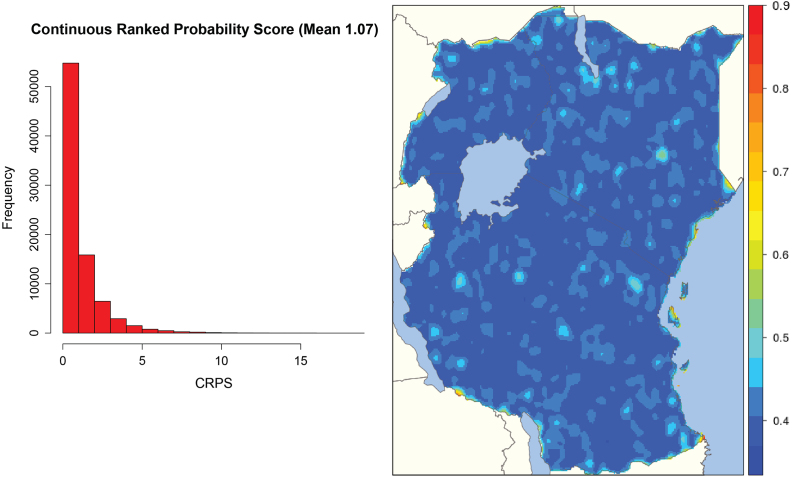
CRPS scores and posterior standard deviation map using our proposed method for the temperature anomaly analysis.

Fig. 6 shows our predicted surface (A) compared with the MRA (B), LatticeKrig (C) and SPDE (D) to the actual data (E). Our final mean absolute error point performance estimates were 3.5 for both our approach and MRA, 3.9 for the SPDE and 4.3 for LatticeKrig. CRPS scores and the posterior standard deviation for our method are shown in Fig. 5. Our model shows strong correspondence to the underlying data and highlights the suitability of using our approach in settings where no relevant covariates exist outside of the spatial coordinates. Our model is thus highly competitive with three of the best performing methods currently available (Heaton et al., 2017). Visual inspection indicates that while the RFF approach captures finer scale detail than both SPDE and LatticeKrig, it is unable to capture fine scale as well as the MRA approach, while having a comparable test score. The observation that RFFs fail to capture very fine scale details has, to our knowledge, never been reported before and deserves future research.

**Fig. 7:**
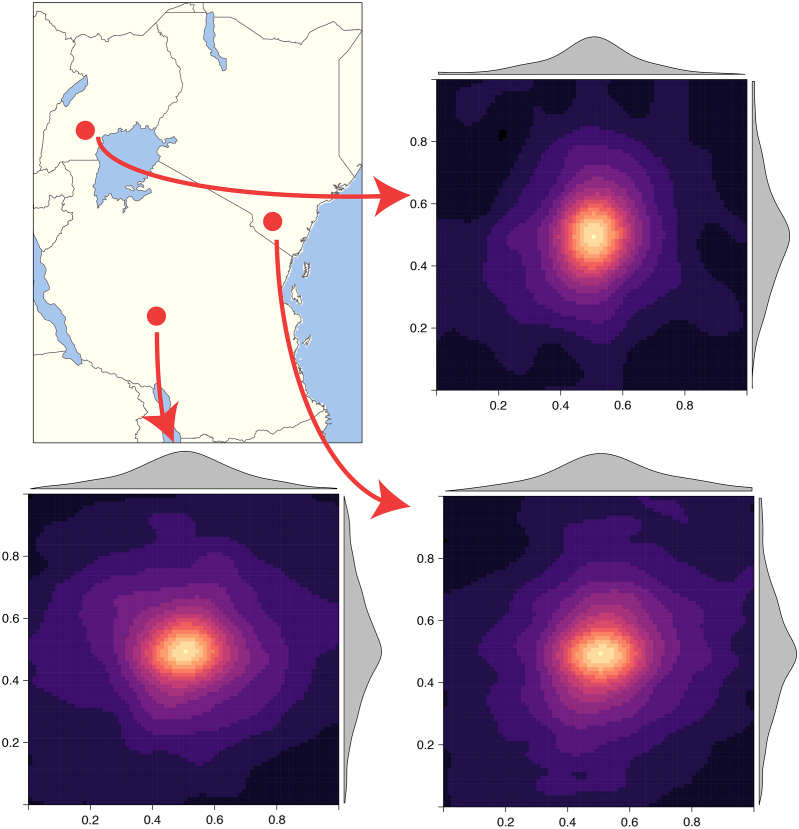
Covariance matrix images for 3 random points showing different covariance structures due to nonstationarity.

**Fig. 8:**
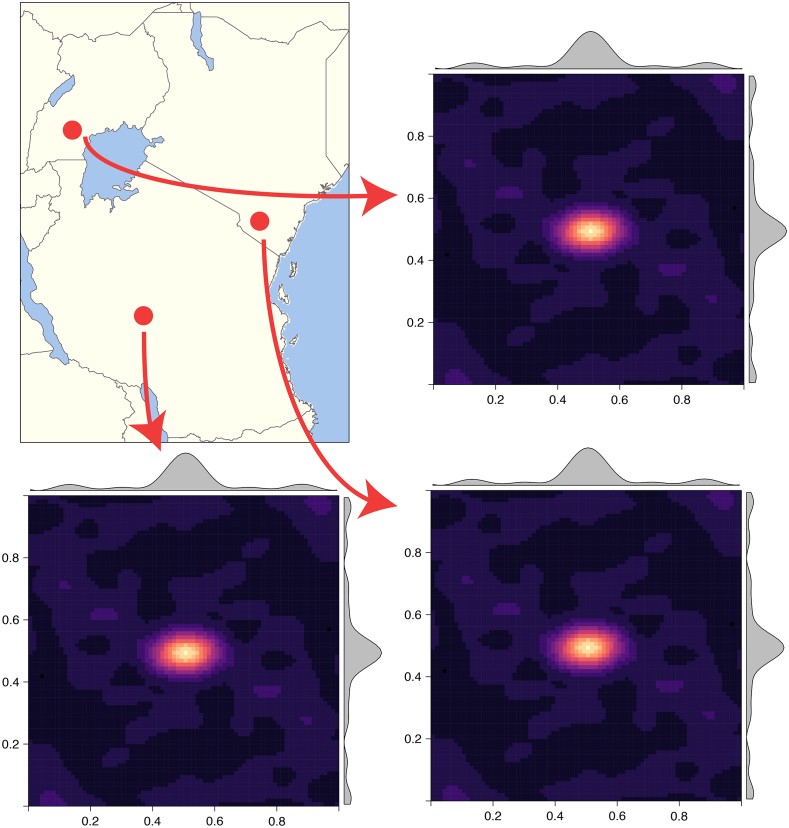
Covariance matrix images for 3 random points showing identical covariance structures for all locations due to stationarity.

Fig. 7 shows 3 randomly sampled points and the covariance patterns around those points. For comparison Fig. 8 shows the equivalent plot when using an RFF stationary squared exponential kernel. In stark contrast to the stationary covariance function, which has an identical structure for all three points, the nonstationary kernel shows considerable heterogeneity in both patterns and shapes. Interestingly the learnt lengthscale/bandwidth seems to be much smaller in the stationary case than the nonstationary case, we hypothesise that this is due to the inability of the stationary kernel to learn the rich covariance structure needed to accurately model temperature anomaly. Intuitively, nonstationarity allows locally dependent covariance structures which conform to the properties of a particular location and imply (on average) larger similarity of nearby outputs and better generalisation ability. In contrast, stationary kernels are trying to fit one covariance structure to all locations and as a result end up with a much shorter lengthscale as it needs to apply to all directions from all locations. Our results are in concordance with other studies showing that temperature anomaly data is nonstationary Remes et al., 2017, Samo and Roberts, 2015, Wu et al., 2007.

Of particular importance is that this interpolation problem can readily be expanded into multiple dimensions including time and other covariates. This is more challenging with the SPDE and MRA approach.

## 5 Discussion

We have shown that nonstationary kernels of arbitrary complexity are as easy to model as stationary ones, and can be learnt with sufficient efficiency to be applicable to datasets of all sizes. The qualitative superiority of predictions when using nonstationary kernels has previously been noted (Paciorek and Schervish, 2006). In many applications, such as in epidemiology where data can be noisy, generalisation accuracy is not the only measure of model performance, and there is a need for models that conform to known biological constraints and external field data. The flexibility of nonstationary kernels allows for more plausible realities to be modelled without the assumption of stationarity limiting the expressiveness of the predictions. It has also been noted that while nonstationary GPs give more sensible results than stationary GPs, they often show little generalisation improvement (Paciorek and Schervish, 2006). For the examples in this work we show clear improvements in generalisation accuracy when using nonstationary kernels. We conjecture that the differences in generalisation performance are likely due to the same reasons limiting neural network performance a decade ago (LeCun et al., 2015) - namely, a combination of small, poor quality data and a lack of generality in the underlying specification. Given more generalised specifications, such as those introduced in this paper, coupled with the current trend of increasing quantities of high quality data (Kambatla et al., 2014) we believe nonstationary approaches will be more and more relevant in spatio-temporal modelling.

There has long been codes of practice on which kernel to use on which spatial dataset (Diggle and Ribeiro, 2007) based on a priori assumptions about the roughness of the underlying process. Using the approach introduced in this paper, *ad hoc* choices of kernel and decisions on stationarity versus nonstationarity may no longer be needed as it may be possible to learn the kernel automatically from the data. For example, our approach can be easily modified to vary the degree of nonstationarity according to patterns found in the data.

In this work we have focused on optimising the marginal likelihood in Gaussian Process regression and added extra regularisation via Gaussian dropout. However, for non-Gaussian observation models the marginal likelihood cannot be obtained in a closed form. In these settings, one may resort to frequentist methods instead and resort to variational (Tan et al., 2013), approximate Rue et al., 2009, Minka, 2001 or suitable MCMC (Carpenter et al., 2017) approaches in order to provide uncertainty measures. For very large models with non-Gaussian observation models, stochastic gradient descent in mini-batches (Bengio, 2012) or stochastic gradient Bayesian methods (Chen et al., 2014) can be used. These Bayesian approaches also address the problem of hyperparameter choice for the Gaussian drop-out and the learning rate parameter.

As with all approximation methods there is a trade off (Bradley et al., 2016) using RFF’s as opposed to other approaches. In general, data-dependent approaches have better convergence bounds better than data-independent approaches when there is a large gap in the eigen-spectrum of the kernel matrix (Yang et al., 2012). However, in contrast to low rank methods, RFFs approximate the entire kernel function directly via sampling from an explicit, lower dimensional, feature map which can improve performance over these methods in some settings (Stein, 2014). It should be noted that the theoretical properties and convergence bounds of RFFs are still not fully understood (Sriperumbudur and Szabo, 2015) and much more research is needed here; as it currently stands the uniform convergence bounds are similar to other approximation methods such as the Nyström (Wu et al., 2016), but simple modifications can greatly improve these bounds e.g. Yang et al. (2016), Yu et al. (2016), Chang et al. (2017) and Chwialkowski et al. (2015). The benefits of RFFs lie in their scalability and ease of implementation coupled with their flexibility in kernel specification that is independent of dimension. In this work we demonstrate that an entire gambit of kernel complexity is possible within the same framework and that predictive performance can be improved in some settings with complex non-stationary kernels.

## Figures and Tables

**Fig. 9:**
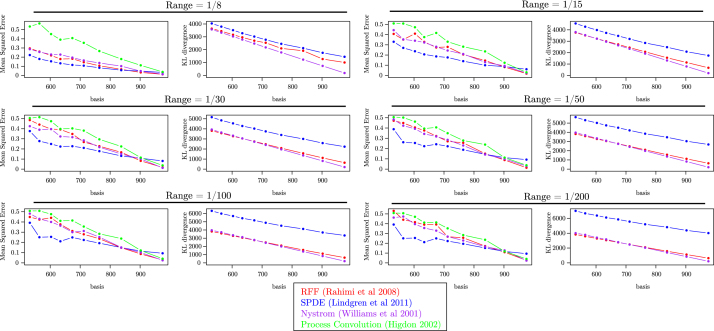
Finite dimensional approximations applied to a full rank Gaussian process of various dependency ranges.

## Appendix Simple performance comparison to commonly used finite dimensional approaches

To provide intuition on the performance of RFFs versus other finite dimensional approximations we perform a simple simulation experiment. A full simulation analysis is beyond the scope of this work and we refer the reader to Bradley et al. (2016) and Heaton et al. (2017).

We simulate a full rank Gaussian process over a spatial two dimensional domain $X\in R^{2}$ and evaluate the performance of 4 commonly used finite dimensional Gaussian process approximations including vanilla RFF’s. The low rank approaches considered are (1) The Nyström Approximation Williams and Seeger, 2001, Rasmussen and Williams, 2006, (2) Lindgren’s stochastic partial differential equation approximation of Lindgren et al. (2011), (3) Higdon’s Process convolutions (Higdon, 2002) and vanilla RFFs (Rahimi and Recht, 2008a). Approaches 1–3 were chosen because of their wide spread adoption in spatial applications Diggle and Ribeiro, 2007, Bhatt et al., 2017, Bhatt et al., 2015, Giorgi and Diggle, 2017, Rasmussen and Williams, 2006, difference in approach, and ease of implementation.

1500 X locations were simulated uniformly over a 1 × 1 plane from a Matérn covariance function of smoothness ν=1 and variance σ=1: $k_{\theta }\left(x_{i},x_{j}\right)=\kappa ∕\tau \|x_{i}-x_{j}\|K_{1}^{\left(2\right)}\left(\kappa \|x_{i}-x_{j}\|\right)$ with $\kappa =\sqrt{2}∕\rho$ an inverse range parameter (for range, ρ), τ a precision (i.e., inverse variance) parameter, and $K_{1}^{\left(2\right)}$ the modified Bessel function of the second kind and order 1. The range ρ, was then varied across $\rho \in \left\{\frac{1}{8}th,\frac{1}{15}th,\frac{1}{30}th,\frac{1}{50}th,\frac{1}{100}th,\frac{1}{200}th\right\}$ spanning a wide variety of plausible long and short range dependencies Bhatt et al., 2015, Measure DHS, 2018.

For all approximations (1)–(4) the primal optimisation problem was solved (linear least squares regression) the mean squared error between the prediction $y^{*}$ and the data y was calculated. Additionally for approximations (1)–(3) the Kullback–Leibler divergence between the full rank, covariance matrix Σ, and the finite dimensional covariance matrix $\Sigma^{*}$ was calculated as $2KL\left(\Sigma ,\Sigma^{*}\right)=tr\left({\left(\Sigma^{*}\right)}^{-1}\Sigma \right)-log\left(|\Sigma |\right)+log\left(|\Sigma^{*}|\right)-n$ (Stein, 2014). The mean squared error measured the approximation quality of the approximated Gaussian process evaluations, and the KL divergence measured the approximation quality of covariance matrix. Note the KL divergence was not calculated for (4) as the method outlined in Higdon (2002) is covariance matrix free.

Fig. 9 shows the results of this experiment. In terms of mean squared error the RFF, SPDE and Nyström approximations are all very similar. The process convolution method is not competitive with the other approaches unless ρ is small and the number of basis functions is large. In terms of KL divergence, the Nyström approximation performs best for all ranges followed by the RFF and SPDE approximations. This simple experiment demonstrates what has previously already been theoretically established; that the RFF approximation is competitive with other widely used covariance approximations Sriperumbudur and Szabo, 2015, Li and Honorio, 2017, Rahimi and Recht, 2009, Rahimi and Recht, 2008a, Rahimi and Recht, 2008b, Avron et al., 2017, Huang et al., 2014, Dai et al., 2014.

In the analysis performed, the computational complexity of (1–4) is not equal. The complexity of the RFF, SPDE and process convolutions is $O\left(nm^{3}\right)$ for n data and m basis functions where as the complexity for the Nyström method is $O\left(m^{3}\right)$ due to the need to perform an additional eigen decomposition step. Additionally, if sparsity was exploited then the SPDE’s complexity can be as low as $O\left(n+m^{\frac{3}{2}}\right)$ which is far superior to the competing methods (Simpson et al., 2015). It should also be noted that the SPDE and process convolution methods are restricted low dimensional settings. Specifically the SPDE approach is restricted to $R^{2}$ problems (and to Matérn, ν=1) and the curse of dimensionality practically limits the process convolution method. The RFF and Nyström methods can model high dimensional Gaussian processes without reductions in error due to dimension (Li and Honorio, 2017).

It should be noted that these experiments are performed using vanilla RFFs and simple adjustments such as performing integration by QMC (Yang et al., 2016), drawing features orthogonally by dimension (Yu et al., 2016), applying shrinkage via the Stein effect (Chang et al., 2017), or dampening the feature expansion with a function Samo and Roberts, 2015, Remes et al., 2017, Chwialkowski et al., 2015 h(x).
